# Fluctuations in episodic and chronic migraine status over the course of 1 year: implications for diagnosis, treatment and clinical trial design

**DOI:** 10.1186/s10194-017-0787-1

**Published:** 2017-10-04

**Authors:** Daniel Serrano, Richard B. Lipton, Ann I. Scher, Michael L. Reed, Walter (Buzz) F. Stewart, Aubrey Manack Adams, Dawn C. Buse

**Affiliations:** 1 Endpoint Outcomes, Boston, MA USA; 2 The Saul R. Korey Department of Neurology, Albert Einstein College of Medicine, Bronx, NY USA; 3 Montefiore Headache Center; Department of Neurology and Department of Epidemiology and Population Health, Albert Einstein College of Medicine, Bronx, NY USA; 4 Department of Preventive Medicine and Biometrics, Uniformed Services University of the Health Sciences, Bethesda, MD USA; 5 Vedanta Research, Chapel Hill, NC USA; 6 Sutter Health, Walnut Creek, CA USA; 7 Allergan plc, Irvine, CA USA; 8 Montefiore Medical Center, Department of Neurology, Albert Einstein College of Medicine, Bronx, NY USA

**Keywords:** Migraine, Chronic migraine, Episodic migraine, Longitudinal, Remission

## Abstract

**Background:**

Relatively little is known about the stability of a diagnosis of episodic migraine (EM) or chronic migraine (CM) over time. This study examines natural fluctuations in self-reported headache frequency as well as the stability and variation in migraine type among individuals meeting criteria for EM and CM at baseline.

**Methods:**

The Chronic Migraine Epidemiology and Outcomes (CaMEO) Study was a longitudinal survey of US adults with EM and CM identified by a web-questionnaire. A validated questionnaire was used to classify respondents with EM (<15 headache days/month) or CM (≥15 headache days/month) every three months for a total of five assessments. We described longitudinal persistence of baseline EM and CM classifications. In addition, we modelled longitudinal variation in headache day frequency per month using negative binomial repeated measures regression models (NBRMR).

**Results:**

Among the 5464 respondents with EM at baseline providing four or five waves of data, 5048 (92.4%) had EM in all waves and 416 (7.6%) had CM in at least one wave. Among 526 respondents with CM at baseline providing four or five waves of data, 140 (26.6%) had CM in every wave and 386 (73.4%) had EM for at least one wave. Individual plots revealed striking within-person variations in headache days per month. The NBRMR model revealed that the rate of headache days increased across waves of observation 19% more per wave for CM compared to EM (rate ratio [RR], 1.19; 95% CI, 1.13–1.26). After adjustment for covariates, the relative difference changed to a 26% increase per wave (RR, 1.26; 95% CI, 1.2–1.33).

**Conclusions:**

Follow-up at three-month intervals reveals a high level of short-term variability in headache days per month. As a consequence, many individuals cross the CM diagnostic boundary of ≥15 headache days per month.Nearly three quarters of persons with CM at baseline drop below this diagnostic boundary at least once over the course of a year. These findings are of interest in the consideration of headache classification and diagnosis, the design and interpretation of epidemiologic and clinical studies, and clinical management.

**Electronic supplementary material:**

The online version of this article (doi:10.1186/s10194-017-0787-1) contains supplementary material, which is available to authorized users.

## Background

Episodic migraine (EM) and chronic migraine (CM) are primarily differentiated by monthly headache days, with EM having fewer than 15 headache days per month and CM having 15 or more headache days per month for at least three months [1]. The epidemiologic profiles of people with EM and CM are well characterized [2–5], as are annual rates of CM remission and CM onset from 1 year to the next [6]. However, relatively little is known about the longitudinal within-person variation in self-reported headache frequency when assessed repeatedly over the course of 1 year.

Migraine can be characterized as a chronic disorder with episodic attacks [7]. Within-person variation in headache days per month logically influences the epidemiologic estimates of EM and CM incidence and persistence reported in the literature [2–5], as well as the assessment of treatment response. However, epidemiology studies, for the most part, have evaluated incidence, persistence, remission, and disease progression by examining only two adjacent points in time. Furthermore, typically, only group mean data are reported. The natural within-person variation in headache day frequency, which is the fundamental driver of the instability in diagnostic classification for EM and CM reported in the literature, has been studied inadequately, if at all. Understanding of this natural history has been obscured in epidemiologic studies because of infrequent repeated measures and the emphasis on aggregated rather than within-person data.

To better characterize within-person change in headache days we analyzed longitudinal data from the Chronic Migraine Epidemiology and Outcomes (CaMEO) Study [8]. We examined transitions from EM to CM and from CM to EM. Of primary importance, we modelled natural history of self-reported headache day variation in quarterly assessments across the span of 15 months. Natural history of headache frequency was modeled accounting for both within-person and between-person changes in headache days per month. These findings have implications for classification and diagnosis, the design and conduct of observational studies, and for the design, management, and interpretation of randomized controlled trials (RCTs).

## Methods

### CaMEO study design and baseline survey

**Fig. 1:**
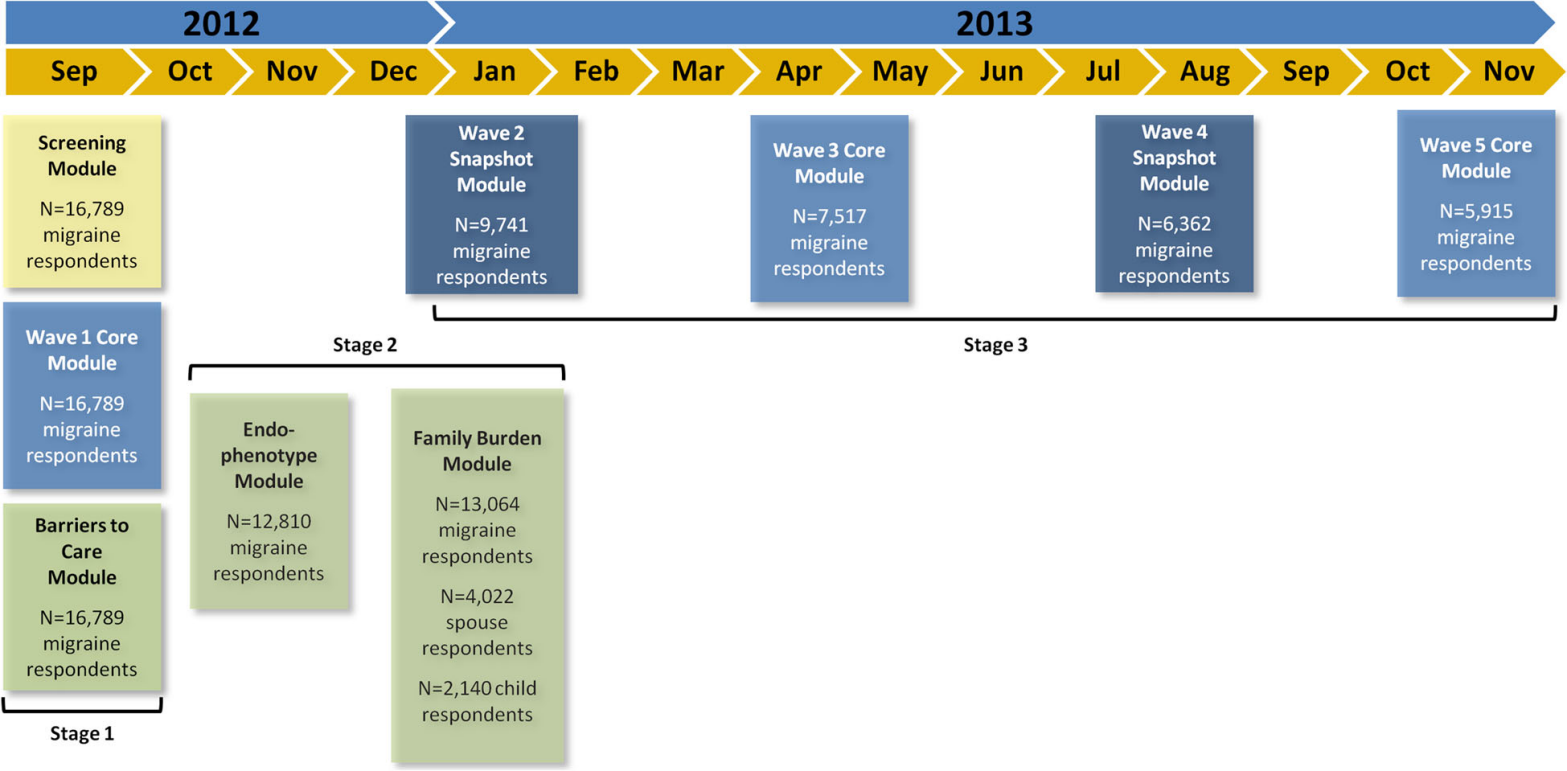
CaMEO study design, reprinted from Manack Adams A, et al. *Cephalalgia* 2015;35:563–578. [8]

The CaMEO Study used a web-based panel (Research Now) constructed to be demographically representative of the US population, as previously detailed [8]. The baseline survey, e-mailed to participants, assessed headache symptoms and severity, headache frequency, headache-related disability, healthcare consulting and utilization, medication use for headache, comorbid medical and psychiatric conditions, and family-related burden associated with headache (Fig. 1).

#### Ethics, consent and permissions

Independent ethics committee approval was obtained before initiation of the CaMEO Study. Given that participation by individuals in the web-based survey was voluntary and that survey research does not expose respondents to known health risks, informed consent was not requested. No person-level identifiable data are included in this study report.

### Study classifications of EM and CM

Respondents were identified as having migraine if they met modified International Classification of Headache Disorders Third edition (beta version) (ICHD-3 [beta]) criteria [1]. Among those with migraine, respondents were classified as having CM if they reported ≥15 headache days per month averaged over the preceding 90 days (i.e., the Silberstein-Lipton approach [9]), otherwise they were classified as having EM. This definition of CM differs from standard ICHD-3 (beta) criteria by not requiring that headaches on eight of the 15 headache days per month be migraine (Criterion C).

### Questionnaire

Of the 58,418 respondents to the baseline survey, 16,789 met criteria for EM or CM and received follow-up surveys at three-month intervals from September 2012 to November 2013. The baseline and four quarterly follow-up assessments constituted the five quarterly waves of data collection in the CaMEO Study, as previously described [8].

Headache frequency was assessed in all five waves using a question from the MIDAS questionnaire about the number of headache days over the last three months [10]. The three-month estimate was rescaled to a monthly estimate by dividing by three, paralleling the approach taken in the American Migraine Prevalence and Prevention (AMPP) study [3]. A diagnosis of EM or CM was assigned at each quarterly assessment. In addition, a continuous measure of headache days per month was retained to model variation in headache days within- and between-persons.

Sociodemographic covariates assessed at baseline included sex (female vs. male), employment status (full-time/part-time employment vs. other), education (college graduate or higher vs. less than college), race (Caucasian vs. non-Caucasian), Hispanic ethnicity (Hispanic vs. not), health insurance status (having health insurance vs. not), household income at or above the national median, and personal income at or above the national median. The two income variables were coded based on median income estimates obtained from the 2012 American Community Survey conducted by the National Census Bureau [11].

In addition, the Migraine Disability Assessment questionnaire (MIDAS) categories were used as a disability covariate, with categories of zero to five indicating no disability (Grade I), six to 10 indicating mild disability (Grade II), 11–20 indicating moderate disability (Grade III), 21–40 indicating severe disability (Grade IV-A), and ≥41 indicating very severe disability (Grade IV-B) [4]. A count of the self-report of physician diagnosis of comorbid conditions was used to generate a comorbidity index; included conditions were asthma, diabetes, high blood pressure, overactive bladder, shingles or herpes zoster, and post-herpetic neuropathy, with a count range of zero (no comorbidities) to six (all comorbidities). Lastly, age at migraine onset reported at baseline and migraine duration (age at baseline minus reported age at migraine onset) were included as disease duration covariates.

The longitudinal wave-specific participation rates for the total sample and stratified by baseline EM and CM status have been presented previously [8]; rates of participation were nearly identical between baseline EM and CM over time.

### Analyses

Analysis first measured longitudinal persistence of baseline EM and CM classification, while accounting for attrition patterns in participation [8]. Analysis was also completed to model variation in average monthly headache days using the longitudinal negative binomial mixed effect models. Orthogonal polynomial terms were used to characterize the longitudinal variation in repeated measures trends and random effects were employed to account for subject-level variance in repeated measures. Technical details of the model building process are available in a separate document. Interested readers may request this document from the first author.

After determining the best-fitting mixed-effect polynomial parameterization, two primary models were fit. The first was an unadjusted model that included only the repeated measures trend terms and main effects and interactions between the repeated measures trend terms and EM/CM status (with EM as reference). The second model adjusted the first with the incorporation of demographic characteristics as well as MIDAS disability, comorbidity count, and disease duration covariates. For the adjusted models, EM and CM status varied over time while all other covariates were entered as predictors based on their baseline status. The GLIMMIX procedure as implemented in SAS (Version 9.2) was used to estimate negative binomial models. All available data were used. Respondents with baseline data only contributed to the estimation of the intercept only. Respondents with more than a single wave of data contributed to estimates of longitudinal trends.

## Results

**Fig. 2:**
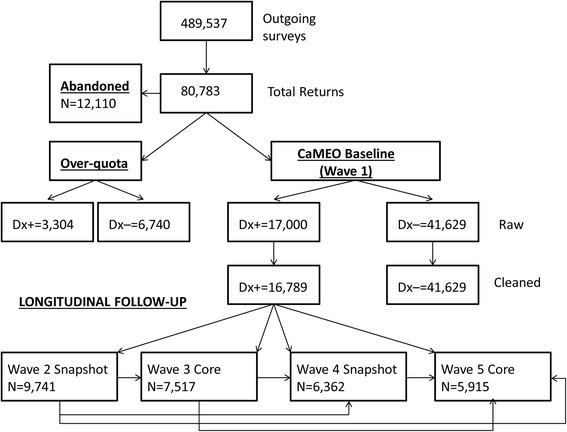
Longitudinal study sample flow

Previously presented data [8] on the sampling design and participation rates will be reviewed to contextualize our findings. Sampling response, attrition, and cleaning rates have been published previously (see Table 2 in Manack et al. 2015) [8]. Of the 16,789 respondents with EM or CM at baseline, (wave one), 10,023 (59.7%) responded and 9741 provided clean and useable returns (58.0% of the baseline sample) at wave two. For subsequent waves, data are available for 7517 participants at wave three, 6363 participants for wave four and 5915 participants at wave five (Fig. 2). The attrition rate decreased with increasing follow-up.

The stability in headache diagnosis was examined descriptively by evaluating the proportion of individuals whose headache diagnosis was maintained across all waves (Table 1). The variation in headache diagnosis was examined by evaluating the proportion of individuals who had at least one wave with the alternative diagnosis. Among the 5464 respondents with EM at baseline providing four or five waves of data, 5048 (92.4%) had EM at all waves and 416 (7.6%) had CM in at least one wave. Among 526 respondents with CM at baseline providing four or five waves of data, 140 had CM at every wave (26.6%) and 386 (73.4%) had EM for at least one wave.

**Table 1:** Baseline headache status stability stratified on waves of study participation

Waves participated	Number of waves baseline headache status maintained	EM at Baseline, n (%)^a^; *n* = 15,313	CM at Baseline, n (%)^a^; *n* = 1476
Respondents participating in 1 wave only (EM, *n* = 4801; CM, *n* = 474)			
1	1	4801 (100)	474 (100)
Respondents participating in any 2 waves (EM, *n* = 2839; CM, *n* = 270)			
2	1	99 (3.5)	159 (58.9)
2	2	2740 (96.5)	111 (41.1)
Respondents participating in any 3 waves (EM, *n* = 2209; CM, *n* = 206)			
3	1	29 (1.3)	98 (47.6)
3	2	98 (4.4)	53 (25.7)
3	3	2082 (94.3)	55 (26.7)
Respondents participating in any 4 waves (EM, *n* = 2161; CM, *n* = 206)			
4	1	12 (0.6)	87 (42.9)
4	2	31 (1.4)	34 (16.7)
4	3	106 (4.9)	34 (16.7)
4	4	2012 (93.1)	48 (23.6)
Respondents participating all 5 waves (EM, *n* = 3303; CM, *n* = 323)			
5	1	21 (0.6)	101 (31.3)
5	2	35 (1.1)	43 (13.3)
5	3	46 (1.4)	32 (9.9)
5	4	165 (5)	55 (17)
5	5	3036 (91.9)	92 (28.5)

*CM* chronic migraine, *EM* episodic migraine

^a^Percentages are the percentages of the respondents participating in the identified number of waves

We modeled headache days per month at three-month intervals using linear, quadratic and cubic polynomial trends for time as well as measures of headache status. In these models, each individual subject is a random effect while the fixed effects represent average trends. As seen in Table 2, relatively large random-effect variances were observed for the polynomial trend terms, including the intercept (0.82), linear trend (0.17), and quadratic trend (0.09), as well as the negative binomial scaling variance parameter (0.09). These random effects reflect the substantial individual variation in monthly headache days. Plots of headache days at an individual-level revealed substantial subject-specific differences and striking curvatures (Additional file 1: Figure. S1). In addition, many fixed-effect estimates were statistically significant. The significant linear trend by headache status interaction indicated that headache days per wave changed differently for those with EM vs CM. Headache days per wave decreased slightly in the EM group and increased slightly in the CM group. This difference in change resulted in a rate of headache day increase of 19% more per wave for CM compared to EM (rate ratio [RR], 1.19; 95% CI 1.13–1.26).

**Table 2:** Unadjusted model for headache days per month for persons with EM and CM

**Random-effect variance parameter estimates**					
	**Estimate**	**Standard Error**			
Intercept	0.82	0.01			
Linear trend	0.17	0.01			
Quadratic trend	0.09	0.01			
Scale	0.09	0.005			
**Fixed Effects**					
**Label**		**RR (95% CI)** ^**a**^	**DF**	**t**	***P*** **value**
Intercept		**3.98 (3.82–4.15)**	16,788	66.53	<0.0001
Linear trend		**0.73 (0.72–0.75)**	29,528	**−**28.49	<0.0001
Quadratic trend		**1.09 (1.06–1.11)**	29,528	7.90	<0.0001
Cubic trend		0.98 (0.97**–**1.00)	29,528	**−**1.51	0.1318
Headache status: CM vs. EM		**4.45 (4.3–4.59)**	29,528	89.36	<0.0001
Headache status by linear trend interaction		**1.19 (1.13–1.26)**	29,528	6.67	<0.0001
Headache status by quadratic trend interaction		1.03 (0.98**–**1.08)	29,528	1.26	0.2075
Headache status by cubic trend interaction		0.99 (0.95**–**1.03)	29,528	**−**0.72	0.4710

*CM* chronic migraine, *DF* degrees of freedom, *EM* episodic migraine, *RR* rate ratio, *t* t-statistic

^a^Statistically significant RRs are bolded

**Fig. 3:**
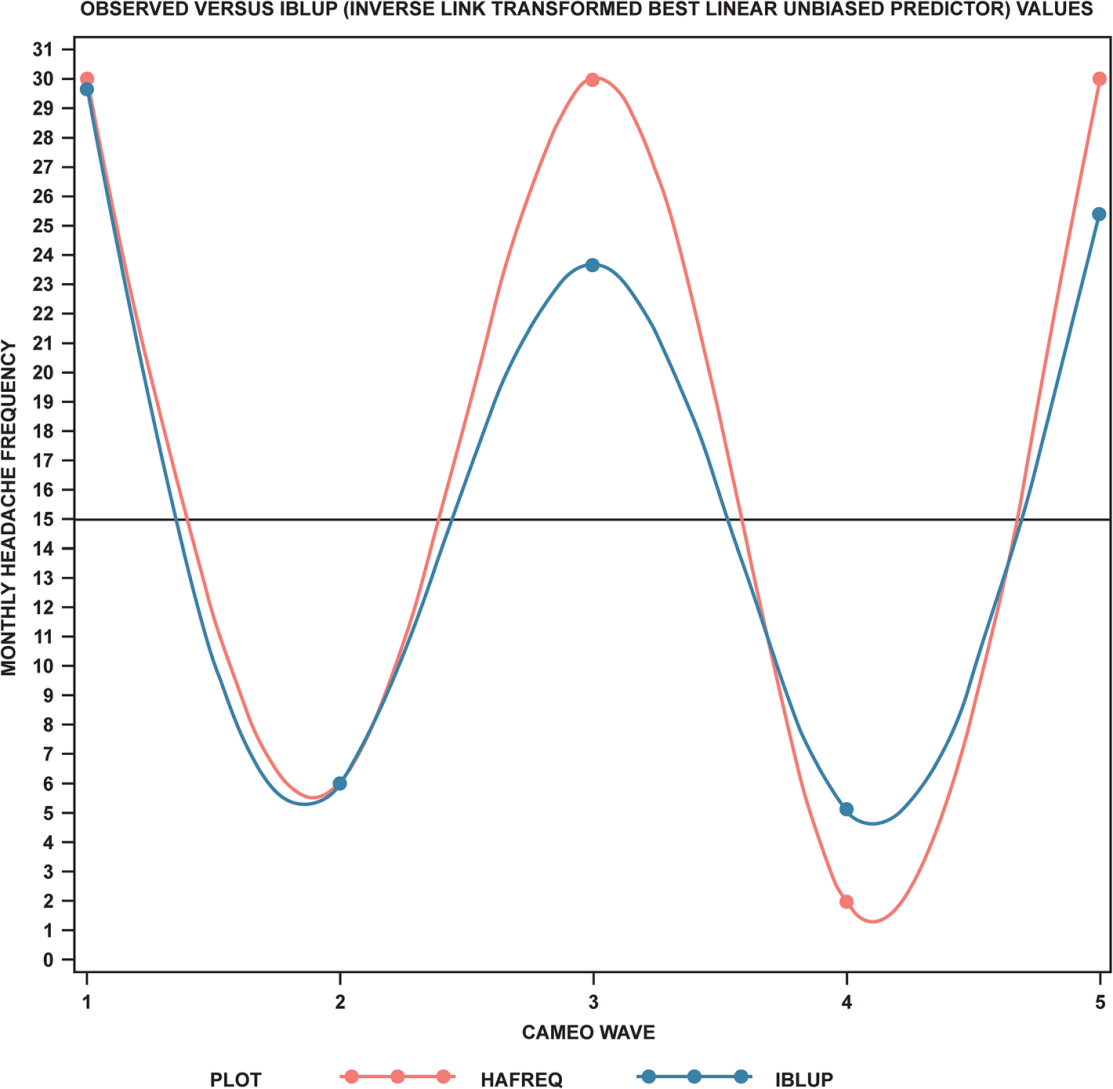
Observed monthly headache frequency over 15 months compared with that predicted using IBLUP = approach HAFREQ = observed monthly headache frequency: IBLUP = inverse link function best linear unbiased predictor

**Fig. 4:**
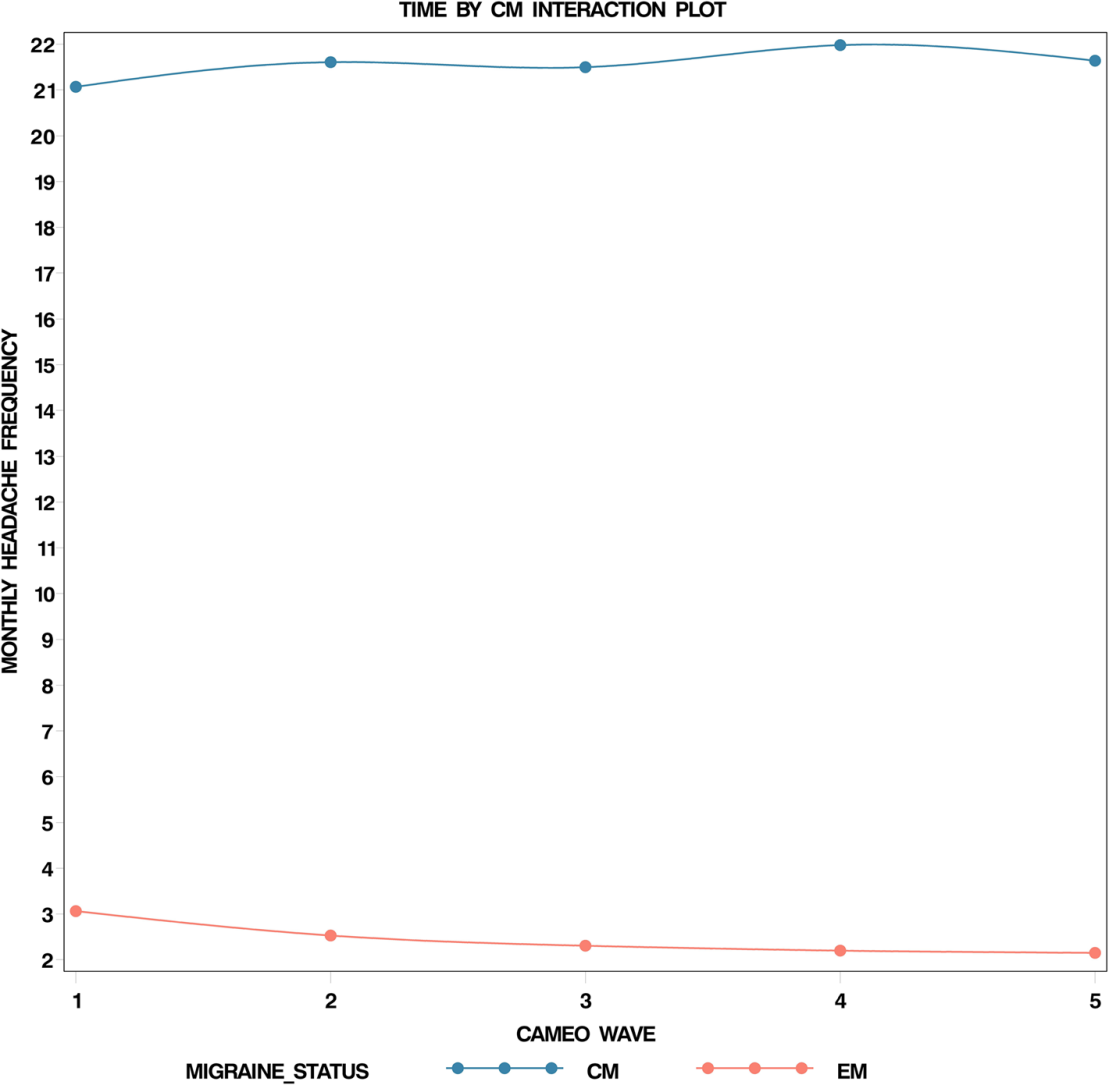
Episodic- and chronic migraine-specific longitudinal trajectories. Time by chronic migraine interaction plot

In Table 3, we augmented the unadjusted model with a set of covariates which diminished the random-effect variance estimates compared with the unadjusted model, particularly the baseline variance (intercept random effect). After adjustment, the random effect variance estimates for the intercept was 0.52, with limited or no reductions in the linear trend variance (0.15), quadratic trend variance (0.09), and the negative binomial scaling variance (0.08) (Table 3). Nearly all fixed effects were statistically significant in the final model. After adjustment, the headache status by linear trend interactions revealed that headache days per month increased 26% more per wave for CM compared to EM (RR, 1.26; 95% CI, 1.20–1.33). Figure 3 demonstrates how closely predicted values reproduced observed variation and oscillation for subjects. Although the predicted values are shrunken toward the mean relative to the observed values, they recover the observed value oscillations quite well. Figure 4 presents a plot of the EM- and CM-specific longitudinal trajectories characterizing this interaction. One can see that headache frequency for EM diminishes over time. In contrast, headache frequency oscillates for CM, but the overall trend is one of subtle increase. The reported rate ratios of 1.19 and 1.26 (adjusted) arise from this difference between headache frequency decreasing over time for EM while increasing over time for CM.

**Table 3:** Final adjusted and trimmed model for headache days per month^a^

**Random-Effect Variance Parameter Estimates**						
	**Estimate**	**Standard Error**				
Intercept	0.52	0.01				
Linear trend	0.15	0.01				
Quadratic trend	0.09	0.01				
Scale	0.08	0.005				
**Fixed Effects** ^**b**^						
** Label**			**RR (95% CI)** ^**c**^	**DF**	**t**	***P*** **value**
Intercept			**1.16 (1.02–1.32)**	16,650	2.28	0.0224
Sex: male vs. female			**0.9 (0.87–0.93)**	16,650	**−**6.62	<0.0001
Education: college graduate vs. non–college graduate			**0.96 (0.93–0.99)**	16,650	**−**2.92	0.0035
Race: Caucasian vs. nonwhite			**1.19 (1.14–1.23)**	16,650	8.78	<0.0001
Household income: at or above national median vs. those below			**0.96 (0.94–0.99)**	16,650	**−**2.42	0.0155
Comorbid count			**1.07 (1.05–1.09)**	16,650	8.06	<0.0001
Disability: MIDAS category TIC			**1.47 (1.46–1.49)**	16,650	73.56	<0.0001
Age at onset, y			**0.998 (0.997–0.999)**	16,650	**−**3.20	0.0014
Duration of illness			1.00 (1.00**–**1.00)	16,650	0.83	0.4090
Linear trend			**0.74 (0.73–0.76)**	29,304	**−**28.63	<0.0001
Quadratic trend			**1.09 (1.07–1.11)**	29,304	8.35	<0.0001
Cubic trend			0.99 (0.97**–**1.01)	29,304	**−**1.34	0.1797
Headache status: CM vs. EM			**3.95 (3.84–4.07)**	29,304	90.71	<0.0001
Headache status by linear trend interaction			**1.26 (1.2–1.33)**	29,304	9.34	<0.0001
Headache status by quadratic trend interaction			0.99 (0.94**–**1.03)	29,304	**−**0.51	0.6434
Headache status by cubic trend interaction			1.00 (0.96**–**1.04)	29,304	**−**0.17	0.8627

*CM* chronic migraine, *DF* degrees of freedom, *EM* episodic migraine, *MIDAS* Migraine Disability Assessment questionnaire, *RR* rate ratio, *t* t-statistic; *TIC* time invariant covariate, *TVC* time-varying covariate

^a^In persons with EM and CM and adjusting for demographic variables, headache related disability and comorbidity

^b^All covariates were specified as TICs, though TVCs could have been specified for several including MIDAS and comorbidity count, which may not have been constant over time

^c^Statistically significant RR are bolded

## Discussion

This study demonstrates that in persons with migraine assessed at three-month intervals, there are frequent transitions between CM and EM with substantial within-person variation in the number of headache days per month over the course of 15 months. Random-effect variance estimates demonstrated that there was significant temporal variability in headache days, both within and between the EM and CM populations. We observed statistically significant increases in headache frequency for CM relative to EM over time, both before and after adjustment for potential confounders. The high rates of transition between EM and CM have implications for headache classification and diagnosis, epidemiologic studies and clinical trial design.

### Implications for headache classification, diagnosis, and clinical practice

We found that 73.4% of people with CM at baseline and four or five follow-up waves of data had at least 1 three-month period when they did not meet the 15 or more headache day per month criteria for CM. Among persons with EM at baseline and four or five waves of follow-up, 7.6% had at least one period when they met the headache frequency criteria for CM. These findings suggest that over the course of 15 months, transitions between EM and CM are very common. In clinical practice, headache diagnosis is often assumed to be relatively stable for an individual, and yet we have found headache frequency changes substantially over time, potentially moving individuals between diagnostic categories (i.e., between EM and CM). When a person transitions from 16 headache days per month to 14 headache days per month, their ICHD-3 (beta) diagnostic label may change but the biology of their disorder most likely does not. The finding that headache day frequency within an individual changes substantially over time poses a considerable challenge to headache classification, diagnosis and subsequently clinical management. As diagnosis is intended to reflect a relatively stable set of clinical features and is used to predict treatment response and clinical course, this level of diagnostic instability is disconcerting. Perhaps headache days alone are not sufficiently stable to be the basis for distinguishing two related nosological entities such as EM and CM. We believe that there are more severe and less severe migraine biological subtypes of migraine, imperfectly differentiated by headache days criteria as EM and CM. Future work could focus on identifying trait predictors of biotype, rather than time-varying features such as headache days per month.

When it comes to interpreting the effects of preventive treatment in practice, we often rely on a short-term reduction in headache days per month. The present findings suggest that at least part of the improvements we attribute to our treatments in practice may represent variation in headache days unrelated to treatment. This hypothesis requires further exploration by examining changes in headache days associated with stable treatment and with changes in treatment.

### Implications for epidemiologic studies

Among people with EM at baseline, when assessed every three months, 7.6% of individuals met headache day criteria for CM at least once over a 15-month period. This is much larger than the 2.5% rate of CM onset in persons with EM we reported after 1 year of follow-up in the AMPP study [12]. This apparent three-fold increased rate of CM onset in persons with EM with more frequent assessments, when coupled with the evidence of transition from CM to EM, suggests that in studies using annual assessments, persons whose CM developed and remitted over the course of the year may be missed. Prior studies have also examined risk factors for CM [3, 13–16] and rates and risk factors for the remission of CM [12, 17, 18]. Previously reported factors associated with an increased risk of CM onset include headache features (such as cutaneous allodynia) [19], a broad array of comorbidities (including depression, allergic rhinitis, and asthma) [15] and poor acute treatment optimization among others [12]. Given the observed relative fluid and repeated movement across the “boundary” of 15 headache days per month in this systematically recruited sample of persons with migraine, the underlying framing of these studies may need to be reconsidered. The defining feature of CM, the number of headache days per month over three months in a person with migraine, may reflect a process of continuous change within a single classification of migraine and not a change in the classification of disease as implied by the previous framing. It is likely that more frequent sampling will identify both higher rates of CM onset and stronger associations between risk factors and this outcome.

### Implications for clinical trial design

Studies of CM use symptom profiles and headache day frequency to determine participation eligibility [20]. In preventive treatment trials of CM, individuals judged by a clinician to have CM typically enter into a four-week diary study prior to study enrollment. If individuals have <15 headache days per month they are deemed ineligible for enrollment into the study. If they have ≥15 headache days per month they are likely to be eligible and offered enrollment into the study. The primary endpoint in prevention trials is typically the change from baseline in the number of headache days per 28-day period on active drug vs. placebo or an active comparator [20]. Change in headache day frequency is typically assessed across two periods (from baseline to follow-up). The spontaneous and rarely measured variation in headache days provides a backdrop for interpreting treatment effects in individuals. There is a substantial probability that persons with CM at baseline will experience reductions unrelated to treatment in headache days as illustrated by the data reported here. Enrolling people with headache days above a threshold may lead to regression to the mean and high “placebo rates” [20]. This in turn can lead to modest therapeutic gains when active drug and placebo are compared [21]. We suggest that the high rate of spontaneous reduction in headache day frequency in RCTs may have contributed to what is widely regarded as an inescapably large placebo effect. This high presumed placebo effect observed in several studies may simply reflect the cyclic nature of CM [22–25].

When the results of our analysis **(**Fig. 3 **)** are considered, one would expect that a majority of subjects selected at any high monthly headache-frequency value would, within three months, have a period of low headache-frequency suggesting that the high placebo rate may be, at least in part, an artifact of inclusion criteria interacting with the natural history of headache frequency. To empirically evaluate this point, we took the data from the CaMEO Study analyzed here, and looked at two waves of data. In the next three paragraphs we demonstrate that by selecting two waves of data and computing change in headache frequency between the two waves, headache frequency decreases for CM in a context where models fit to all waves demonstrated that frequency actually increased for CM. This calls into question the interpretability of two time-point change computations for headache frequency outcomes in both epidemiology studies and clinical trials.

Change in one-month recall of headache frequency was compared across two pairs of complete data from the CaMEO Study waves. The first pair was composed of waves one and two, which, with three months between them, directly mimics the assessment interval for the placebo arm in many prophylaxis trials. The second pair assessed longer-term change and was composed of waves one and five. Two variables were considered: (1) raw headache frequency and (2) change in headache frequency (headache frequency reported in the follow-up wave minus that reported in wave one). The effect of interest was whether change in headache frequency across the waves was different for EM and CM. Descriptive statistics, including wave EM- and CM-specific sample sizes and means for these three variables are given in Table 4. For the wave-specific headache frequency variables, a repeated measures negative binomial model was parameterized to calculate the difference between waves and estimate the difference in this difference between EM and CM through a time-by-headache status interaction term. The resulting time-by-headache status interaction RR emerging from this model along with the corresponding 95% CI and *P* values are reported in Table 4. This model was employed because it matches the modeling framework for the other models reported in this paper. For the variable in which change from baseline was calculated, a simple one-way analysis of variance (ANOVA) was conducted in which the only effect was baseline headache status. This model estimated the mean difference in change in headache frequency between baseline and follow-up between EM and CM, treating EM as the reference group. The resulting mean difference and corresponding 95% CI and *P* values are reported in Table 4.

**Table 4:** Approximation of placebo-arm findings in RCTs as a result of “natural” remission of CM

	EM				CM				Negative Binomial Mixed-Model for Change From Baseline to Follow-Up^e^			1-Way ANOVA for Headache Frequency Change Score From Baseline to Follow-Up	
Time Contrast		Baseline	Follow-Up^b^	Change Score		Baseline	Follow-Up^b^	Change Score	Baseline Headache Status by Time Interaction Random-Effect Model-Based			Baseline Headache Status ANOVA Effect	
	Sample size^a^	μ_1_	μ_2_^d^	μ_Δ_	n	μ_1_	μ_2_^d^	μ_Δ_	Raw^f^	RR	*P* value	Mean Difference^c^	*P* value
Wave 1 vs. Wave 2	7759	3.98	4.14	0.16	829	19.77	14.61	−5.16	0.71	0.67 (0.64 to 0.71)	<0.0001	−5.32 (−5.69 to −4.95)	<0.0001
Wave 1 vs. Wave 5	4407	4.05	4.09	0.05	502	19.80	13.69	−6.12	0.68	0.64 (0.59 to 0.69)	<0.0001	−6.17 (−6.69 to −5.64)	<0.0001

*ANOVA* analysis of variance, *CM* chronic migraine, *EM* episodic migraine, *HA* headache, *RCT* randomized controlled trial, *RR* rate ratio, *SAS* statistical analysis system

^a^Sample sizes were based on complete case data, and are therefore lower for the second time contrast than the first because less subjects chose to respond to the survey by wave 5 than wave 2. The combined sample sizes are less than the wave 2 or wave 5 sample sizes respectively (i.e., 4407 + 502 = 4909, which is <5915) because the complete case data restriction also required the same subjects contribute data on the past month HA frequency variable at both the baseline and follow-up assessments

^b^Baseline Headache status by time interaction estimation: (CM_ μ_2_/ CM_ μ_1_)/(EM_ μ_2_/ EM_ μ_1_)

^c^Baseline Headache status ANOVA mean difference: CM_ μ_Δ_ – EM_ μ_Δ_

^d^Random-effect model-based estimate parameterized with a random subject-specific intercept, fixed intercept, main effect for headache status (EM vs. CM, EM reference), main effect for time contrast (baseline vs. follow-up, baseline reference), and the interaction between headache status and time

^e^Random-effect negative binomial models were fit in SAS’ GLIMMIX procedure. The model estimation was identical to the primary models presented in this manuscript and described in the methods section with adaptive Gauss-Hermite quadrature based on 13 quadrature points and 9 pseudo-likelihood initial iterations for start values, with no generalized linear model–based iterations

^f^Raw means, raw RRs, and random-effect model-based rate ratios indicate that HA frequency for CM declines over time while HA frequency for EM remains stable. Moreover, CM declines more as the amount of time between assessments increases (2 waves vs. 5). Specifically, with only three months between assessment, HA frequency declines 33% more for CM than EM (100*[1–0.67]), while HA frequency declines 36% more for CM than EM (100*[1–0.64]) when the time between assessments is 12 months

In both cases (wave one and two pairing or wave one and five pairing), the results were identical. It appears that no matter what pairing of waves is used, headache frequency significantly decreases for CM, while headache frequency for EM remains mostly constant. The change in headache frequency for CM was so great that at follow-up the average headache frequency would have resulted in the average population being classified as having achieved CM remission in both wave pairings through the natural history of the condition alone. The reported RRs for the time-by-baseline headache status interaction and the ANOVA mean difference support this conclusion and indicate that the CM reduction in headache frequency between wave pairings was statistically significantly greater than that of EM.

Results presented here suggest that when subject selection is made on the basis of high frequency of headache days at baseline, as a direct consequence of the cyclical nature of monthly headache day frequency, there is a nontrivial probability that within a short period of time, headache frequency will organically fall to lower levels. However, and most importantly, headache day frequency does not remain low, and for a sizeable number of people, headache frequency values return to even higher levels. This appears as a fundamental paradox: in the full data headache frequency for CM increases over time while comparisons between pairs of waves suggest that headache frequency decreases for CM. This paradox is related to a variant of the Yule-Simpson effect [26], where conclusions are distorted by specific binning or grouping of the data that is not representative of the true global trend in the phenomenon. Results presented in this manuscript would suggest that variation in headache frequency is substantial. Moreover, the practice in RCTs of restricting attention to a baseline period obtained through selection based on a high frequency of headache days in the baseline period coupled with examination of only a single subsequent follow-up period leads to the frequently observed placebo effect for CM prophylaxis trials. The use of more sensitive and sophisticated designs and analyses may enable more accurate characterization of longitudinal headache epidemiology and determine with greater certainty the efficacy of treatment.

### Strengths and limitations

This study has a number of strengths and limitations. Strengths include the large sample size with inclusion of large numbers of persons with migraine, the three-month follow-up interval over a period of 15 months and our well-accepted modeling strategy. Limitations include the modest participation rate and the relatively high drop-out rate from wave to wave. The modest participation rate is offset in part by the evidence that results from the CaMEO Study are largely comparable to those from the larger AMPP study [6] and the non-responder study that did not provide any suggestion of participation bias [8]. As headache frequency varies over longer periods, modeling headache days using control theory–based models may provide better long-term characterization of the natural history of migraine across multiple intervals. Furthermore, the dynamic nature of migraine (transitioning between EM and CM) needs to be considered when a categorical description (i.e., EM or CM) is captured at baseline or individual time points. For example, a person categorized as having EM at baseline may have been classified with CM previously.

In addition, this study used modified ICHD-3 criteria which did not confirm ≥ five lifetime migraine events (criterion A) or duration of attack untreated from four to 72 h (criterion B). Likewise, the definition of CM did not include criterion C which states that migraine occurred ≥ 8 days per month. Confirmation of these parameters is difficult to evaluate via self-report retrospective surveys and requires data collection via diary cards and physician interview to verify.

## Conclusions

Results confirm that there is substantial variation in headache day frequency in people with EM and CM followed at three-month intervals. Transitions from EM to CM are more common (7.6%) than previously observed when sampled less frequently; in addition, nearly 75% of people with CM will remit to EM at some point during a 12-month period. More research is required to more fully understand the implications of these findings. Importantly, this natural variation should be considered when designing epidemiologic and clinical trials and when clinicians diagnose, treat and study CM.

## Additional file

Additional file 1: Figure S1. Variability of Headache Day Frequency for Individuals with A) Episodic Migraine and B) Chronic Migraine at Wave One. (PDF 1392 kb)

## Abbreviations

AMPP: American Migraine Prevalence and Prevention
ANOVA: Analysis of variance
CM: Chronic migraine
EM: Episodic migraine
IBLUP: Inverse link function best linear unbiased predictor
ICHD-3 (beta): International Classification of Headache Disorders Third edition (beta version)
MIDAS: Migraine Disability Assessment questionnaire
RCT: Randomized controlled trial
RR: Rate ratio
SAS: Statistical analysis system
