# Supplementary material for: Fluctuations in episodic and chronic migraine status over the course of 1 year: implications for diagnosis, treatment and clinical trial design
Source: J Headache Pain. 2017 Oct 4;18(1):101. doi: 10.1186/s10194-017-0787-1 (PMC5628086; doi:10.1186/s10194-017-0787-1)
Supplement: Additional file 1: Figure S1. — Variability of Headache Day Frequency for Individuals with A) Episodic Migraine and B) Chronic Migraine at Wave One. (PDF 1392 kb) [file 10194_2017_787_MOESM1_ESM.pdf]

Supplementary Figure 1. Variability of Headache Day Frequency for Individuals with A. Persons with CM had baseline who remit in Wave 2 or Wave 3. B. Persons with CM at baseline who remit in Wave 2 C. Persons with EM at baseline CM during follow-up

A.

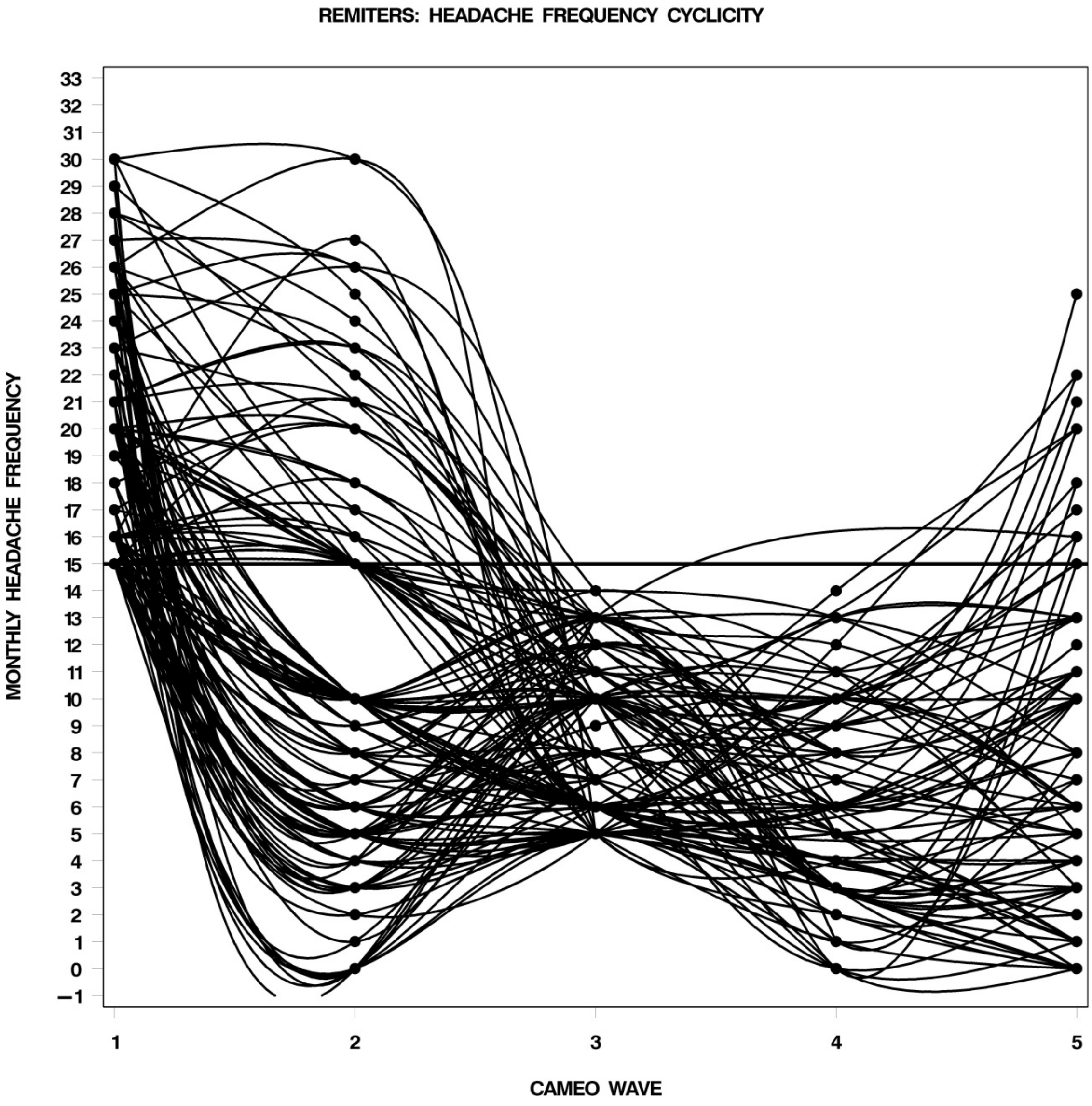

B.

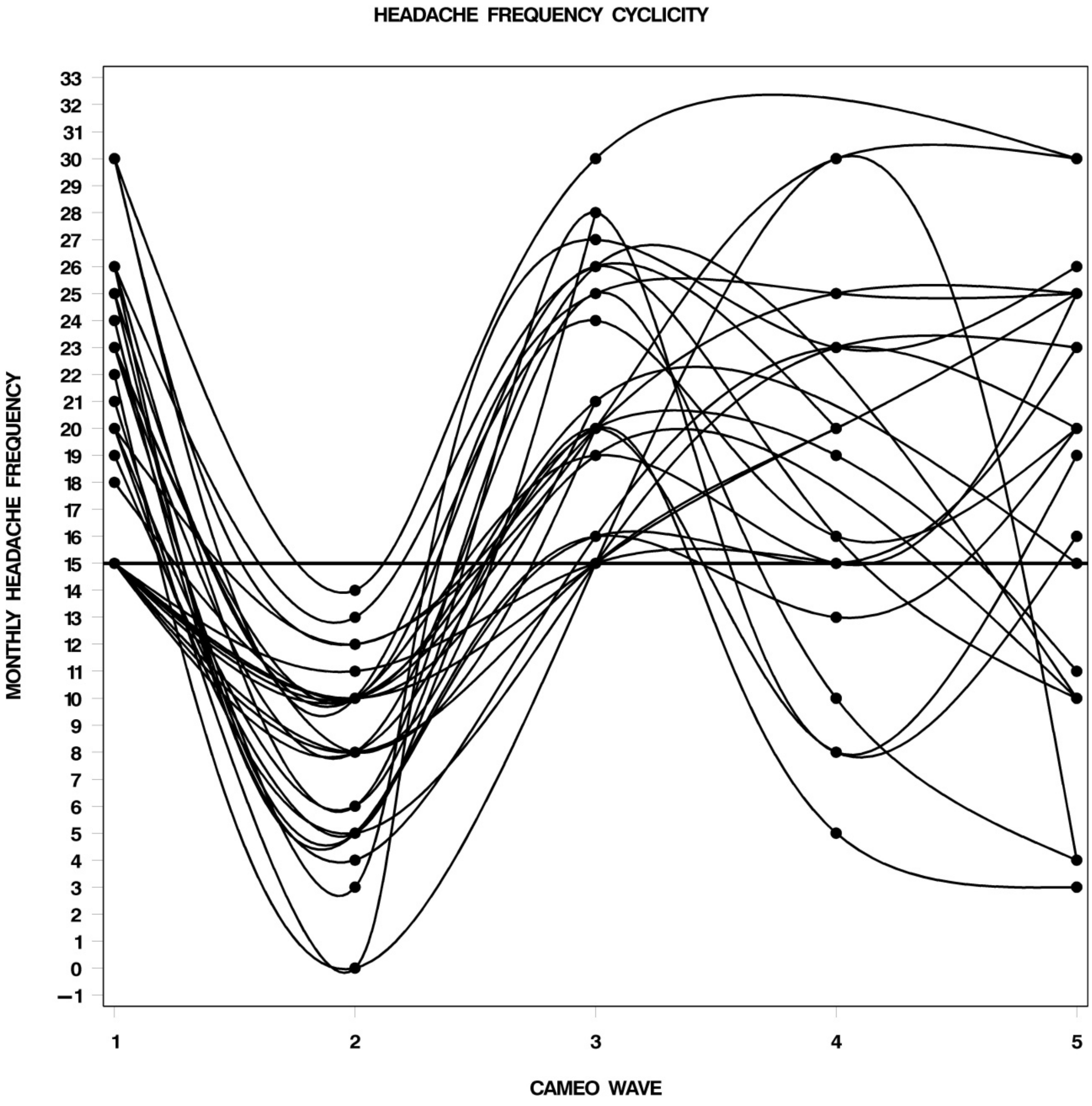
HEADACHE FREQUENCY CYCLICITY
MONTHLY HEADACHE FREQUENCY
33
32
31
30
29
28
27
26
25
24
23
22
21
20
19
18
17
16
15
14
13
12
11
10
9
8
7
6
5
4
3
2
1
0
−1
1
2
3
4
5
CAMEO WAVE

C.

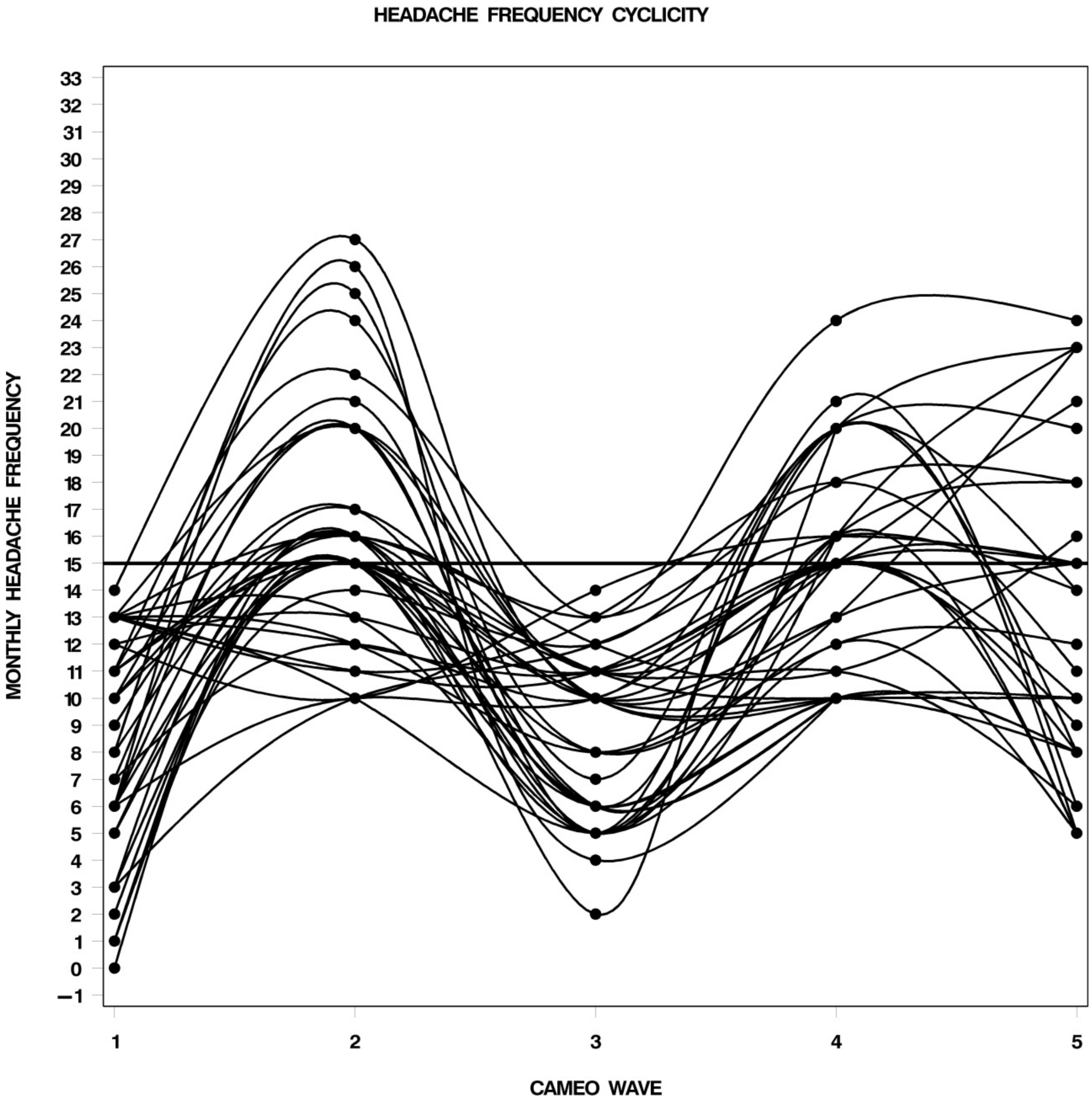
HEADACHE FREQUENCY CYCLICITY
MONTHLY HEADACHE FREQUENCY
CAMEO WAVE
33
32
31
30
29
28
27
26
25
24
23
22
21
20
19
18
17
16
15
14
13
12
11
10
9
8
7
6
5
4
3
2
1
0
−1
1
2
3
4
5
